# Increased levels of anti-*Encephalitozoon intestinalis* antibodies in patients with colorectal cancer

**DOI:** 10.1371/journal.pntd.0012459

**Published:** 2024-09-09

**Authors:** Céline Nourrisson, Maxime Moniot, Leslie Vercruysse, Virginie Bonnin, Bruno Pereira, Nicolas Barnich, Mathilde Bonnet, Marine Jary, Denis Pezet, Johan Gagnière, Philippe Poirier

**Affiliations:** 1 Parasitology-Mycology Department, CHU Clermont-Ferrand, 3IHP, Clermont-Ferrand, France; 2 Clermont Auvergne University, « Microbes, Intestin, Inflammation et Susceptibilité de l’Hôte » M2iSH, UMR INSERM 1071, INRAe 1382, Clermont-Ferrand, France; 3 National Reference Center (NRC) for cryptosporidiosis, microsporidia and other digestive protozoa, Clermont-Ferrand, France; 4 Biostatistics Unit, DRCI, CHU Clermont-Ferrand, Clermont-Ferrand, France; 5 Digestive Oncology Department, CHU Clermont-Ferrand, Clermont-Ferrand, France; 6 Digestive Surgery Department, CHU Clermont-Ferrand, Clermont-Ferrand, France; Institute of Continuing Medical Education of Ioannina, GREECE

## Abstract

**Background:**

The prevalence of microsporidiosis in the general population, or within specific groups of individuals/patients, is largely underestimated. The absence of specific seroprevalence tools limits knowledge of the epidemiology of these opportunistic pathogens, although known since the 1980s. Since microsporidia hijack the machinery of its host cell and certain species multiply within intestinal cells, a potential link between the parasite and colorectal cancer (CRC) has been suggested.

**Methodology/principal findings:**

To explore a potential epidemiological link between microsporidia and CRC, we evaluated the seroprevalence of *Encephalitozoon intestinalis* among CRC patients and healthy subjects using ELISA assays based on two recombinant proteins, namely rEiPTP1 and rEiSWP1, targeting polar tube and spore wall proteins. ELISA were performed in 141 CRC patients and 135 healthy controls. Patients with CRC had significantly higher anti-rEiPTP1 IgG levels than subjects in the control group. Anti-rEiPTP1 IgG, anti-rEiSWP1 IgG and anti-rEiPTP1 IgA levels were significantly increased among men with CRC compared to healthy men. Women with CRC who had died had higher rEiSWP1 IgG levels than those who were still alive.

**Conclusions/Significance:**

These higher antibody levels against microsporidia in patients with CRC suggest a relationship between microsporidia and pathophysiology of CRC.

## Author summary

The role of infectious agents in the occurrence of cancer is increasingly studied. Microsporidia are pathogens that can cause intestinal damage leading to severe diarrhea in humans. Data previously published would allow to consider a role of microsporidia in the occurrence of colorectal cancer. Our objective here was to provide epidemiological data concerning the association of microsporidia and cancer, which will be additional arguments that can justify in-depth work to study a potential causal link. To do this, we have developed tests to detect antibodies directed against a species of microsporidia, *Encephalitozoon intestinalis*. Our results show higher levels of antibodies against *E*. *intestinalis* among patients with colorectal cancer, compared to individuals without cancer, particularly in men. We also found that levels of certain antibodies were lower in women who survived cancer compared to those who died, possibly suggesting that this is a prognostic marker. All of these results support an epidemiological link between microsporidia, or at least certain species, and colon carcinogenesis.

## Introduction

Microsporidia are intracellular eukaryotes mainly responsible for intestinal infections in humans [1]. These cause profuse watery diarrhea and abdominal pain. *Enterocytozoon bieneusi* is by far the most common among the species involved, followed by *Encephalitozoon intestinalis* and *Encephalitozoon hellem* [1,2]. Infection follows the ingestion of microsporidia spores present in contaminated water or food, but human-to-human contamination is also suspected [1].

*In vitro* data about the intracellular impact of *E*. *bieneusi* infection are not available because it is still uncultivable until now. However, it is nowadays well admitted that microsporidia hijack the host’s cellular machinery. Thus, experimental studies on other species, such as those belonging to *Encephalitozoon* genus, have demonstrated that microsporidia exert an effect on the cell cycle and apoptosis, both mechanisms being involved in cancer pathophysiology [3–5]. Furthermore, some studies showed a significantly higher detection rate of microsporidia (*E*. *intestinalis*, *E*. *bieneusi*) in stools of cancer patients than in those of a healthy population [6,7]. However, it is unclear whether microsporidia are involved in cancer pathogenesis or whether cancer-induced immunosuppression promoted infection. Moreover, the absence of detection of microsporidia cannot exclude a previous infection which resolved spontaneously and which could have participated in cancer initiation. Thus, serological analyses could be more accurate to assess the real burden of microsporidia infections in cancer patients.

Few studies have used serological tools targeting microsporidia. They were essentially based on immunofluorescence techniques on whole spores, or on ELISA assays using total protein extracts from spores produced *in vitro*, or even, more rarely, on recombinant proteins [8–11]. In a recent publication, Redondo et *al*. observed an increase in both anti-*Encephalitozoon cuniculi* IgG and IgE antibodies levels in colorectal cancer (CRC) patients, regardless of gender or age, and suggested that the presence of the parasite predates the cancer diagnosis [12]. This ELISA assay was performed on total antigens from *E*. *cuniculi* spores in culture, obtained after extraction with detergent and reducing agents. *E*. *cuniculi* is responsible for rare infections in humans and then causes hepatitis, endocarditis or disseminated infections. Another species of the *Encephalitozoon* genus, namely *Encephalitozoon intestinalis*, is more frequently detected in humans and has been associated with diarrhea [1].

During an infection by a microsporidia, a particular structure coiled within the spore, called the polar tube, will be extruded and form a hollow tube allowing the transfer of genetic material into the host cell [1]. Also, following contamination by the *Encephalitozoon* genus, the synthesis of antibodies directed against spore wall proteins (SWPs), initially, then against polar tube proteins (PTPs), secondly, has been described [8].

Thus, we developed ELISA assays based on both crude antigens and specific recombinant proteins (SWP1 and PTP1) from *E*. *intestinalis*. These tests were applied to a cohort of colorectal cancer (CRC) patients and to a cancer-free control population.

## Methods

### Ethics statement

The study was approved by local Ethics Committee (IRB00013412, “Centre Hospitalier Universitaire de Clermont Ferrand IRB #1”, IRB number 2024-CF326) with compliance to the French policy of individual data protection. An information note was sent to the included subjects, who could express their opposition in return, the absence of a response was considered consent.

### Study population

CRC patients treated in the digestive surgery department of the University Hospital of Clermont-Ferrand (France) between 2013 and 2017 were included. A group of control patients, hospitalized in the same facility, without known cancer or immunosuppression history, was formed, correlated in age and sex with the CRC patients. The informations available for the CRC group were: tumor location, history of chemotherapy before surgery, international TNM classification [13] (tumor, node, metastase), presence of metastases, relapse and death. For each patient, plasma was collected and stored at -80°C until use. The number of freeze-thaw cycles of the samples was controlled by the storage of small volume aliquots, so each sample used did not undergo more than two thawings.

### Microsporidia culture

*E*. *intestinalis* (ATCC 50506), *E*. *cuniculi* (ATCC 50602) and *E*. *hellem* (isolated from patient stool and previously axenised by us [2]) were maintained in culture in rabbit kidney RK-13 cells (ATCC CCL-37) at 37°C with 5% CO_2_ using Minimum Essential Medium (MEM, Gibco) supplemented with 5% fetal calf serum, 2 mM of L-glutamine (Eurobio Scientific), 10000 U/mL of penicilline, 10 mg/mL of streptomycin and 25 μg/mL of amphotericin B (Cytiva). Microsporidia culture supernatants, containing spores, were collected three times a week and stored at 4°C until use.

### Crude antigens of *E*. *intestinalis*

Spores of *E*. *intestinalis* were centrifuged at 1300 g for 10 min at 4°C, then the pellet was resuspended in distilled water. After three washes with PBS, the pellet was resuspended in a lysis buffer containing 50 mM of Tris-HCl at pH 7.5, 150 mM of NaCl, 2.5% of SDS, 100 mM of DTT, 1% of Nonidet P-40, 20 mM of EGTA and a cocktail of proteases and phosphatases inhibitors (Halt Protease and Phosphatase Inhibitor Cocktail, ThermoFisher Scientific). A bead beating step was performed by adding a mix of lysing beads (MP Lysing Matrix E, MP Biomedicals) to the suspension, followed by disruption at 30 Hz for 3 min in Tissue Lyzer (Qiagen) at 4°C. After centrifugation, the supernatant was boiled for 15 min. Protein quantification was performed with the DC Protein Assay (Bio-Rad). Protein extract was stored at -20°C until use.

### Recombinant proteins

*E*. *intestinalis* DNA was extracted from the *E*. *intestinalis* ATCC 50506 strain. PCR was performed to amplify the genes encoding polar tube protein 1 (PTP1) and spore wall protein 1 (SWP1) as previously described [14,15]. In all cases, forward and reverse primers contained one *Bam*HI and one *Xho*I restriction site at their 5’ end, respectively. PCR reactions were performed according to standard conditions (High-Fidelity DNA polymerase) with an annealing step at 50°C. PCR products were then cloned into the pGEX-4T-1 expression vector modified by the addition of a 6-His tag and recombinant plasmids were sequenced. Each recombinant plasmid was introduced into *E*. *coli* BL21 Codon Plus strain (Agilent) and protein production was induced with 0.5 mM IPTG, bacteria were grown overnight at 16°C with 100 μg/mL ampicillin. After centrifugation at 4000 g for 10 min at 4°C, cells were disrupted by sonication at 4°C in a lysis buffer containing 50 mM NaH_2_PO_4_ pH 8, 300 mM NaCl, 5 mM EDTA and 1 mg/mL lysozyme. Protein pellets were washed three times in a buffer containing 50 mM NaH_2_PO_4_ pH 8, 300 mM NaCl, 5 mM EDTA and 3 M urea. Recombinant proteins were solubilized under denaturing conditions in urea buffer (8 M urea, 50 mM NaH_2_PO_4_ pH 8, 300 mM NaCl) for 30 min at 4°C under agitation. Recombinant proteins were purified using nickel-charged resin (Ni-NTA Agarose, Qiagen) according to the manufacturer’s recommendations. Elution was done at 4°C in 50 mM NaH_2_PO_4_, 300 mM NaCl pH 8 containing 500 mM imidazole. Imidazole was then removed by centrifugation on Amicon Ultra 10 K (Millipore) at 4°C. Protein purity was assessed by 10% SDS-polyacrylamide gel electrophoresis. Protein dosage was performed with the DC Protein Assay (Bio-Rad). Recombinant proteins were stored at -20°C until use. The recombinant PTP1 of *E*. *intestinalis* was named rEiPTP1, and the recombinant SWP1 was named rEiSWP1.

### Mouse immunization

Polyclonal antibodies directed against rEiPTP1 and rEiSWP1 were obtained by mouse immunization. Briefly, purified recombinant proteins were inoculated intraperitoneally into C57BL6/J mice, according to the following protocol: a primary injection followed 15 days later by a weekly booster injection for 4 weeks. Then mice were sacrificed and blood was collected. Then serum was obtained after centrifugation at 10,000 g for 5 minutes. Serum were stored at -20°C until use. The experiments were performed in accordance with the ethical guidelines set out in the Guide for the Care and Use of Laboratory Animals of the University of Clermont Auvergne and were approved by the French Ministry of National Education, Higher Education and Research (APAFIS #44542).

### Immunofluorescence assay

In order to evaluate the polyclonal antibodies pAb-rEiSWP1 and pAb-rEiPTP1 specificity, an indirect immunofluorescence technique (IFAT) was performed on spore smears of *E*. *intestinalis* in culture. Between each of the following steps, slides were washed three times in PBS– 0.05% Tween 20 (PBS-T). After a blocking step with 5% (m/v) powdered milk in PBS-T at room temperature overnight, anti-rEiPTP1 mouse polyclonal antibody diluted at 1/200 in 1% powdered milk—1% Tween 20 –PBS was incubated for 2 h. Then, Alexa Fluor 488 conjugated goat anti-mouse IgG (Cell Signaling) diluted at 1/100 was incubated for 1 h. Anti-rEiSWP1 mouse polyclonal antibody diluted at 1/200 in 1% powdered milk—1% Tween 20 –PBS was added and incubated for 2 h. Then, Alexa Fluor 647 conjugated goat anti-mouse IgG (Cell Signaling) diluted at 1/100 was incubated for 1 h. Finally, the mounting liquid was added.

In order to verify results obtained with our ELISA assay, IFAT were performed on spore smears of *E*. *intestinalis* (*in vitro* culture, ATCC 50506). The steps were the same as those described above, except for primary antibody which was the plasma of patients considered positive or those of patients considered negative (*i*.*e*. patients with *E*. *intestinalis* intestinal microsporidiosis previously diagnosed in our laboratory, or patients whitout known history of this infection, respectively) were used as primary antibody, and the secondary antibody was a FITC conjugate goat anti-Human IgG (Bethyl). DAPI (ThermoFisher Scientific) diluted at 1/100 in PBS was added and incubated for 5 min. Then the mounting liquid was added.

### Western blot

Spores of *E*. *intestinalis*, *E*. *cuniculi* and *E*. *hellem* were collected from culture supernatants and concentrated by centrifugation (1300 g for 10 min). The pellets were mixed with 0.5 mm glass beads and grinded with Tissue Lyser (Qiagen) at 30 Hz for 3 min. After centrifugation (10000 g for 5 min), the pellets were resuspended in Laemmli sample buffer and boiled for 5 min. Insoluble material was removed by centrifugation, and cleared supernatants were used for migration in polyacrylamide gel. Proteins were transferred onto PVDF membranes. Between each of the following steps, membrane was washed three times in PBS– 0.1% Triton (PBS-Tr). The membrane was incubated for 1 h at room temperature with a blocking solution containing 5% (m/v) powdered milk in PBS-Tr. Then anti-rEiPTP1 or anti-rEiSWP1 mouse polyclonal antibodies diluted at 1/1000 were incubated at 4°C overnight. Then horseradish peroxydase conjugated horse anti-mouse IgG (Cell Signaling) diluted at 1/2000 was incubated for 1 h. The revelation was performed with the Clarity Western ECL Substrate (Bio-Rad) and signals were observed with the ChemiDoc Imaging System (Bio-Rad).

### ELISA assay

ELISA methods were designed using the *E*. *intestinalis* crude antigen extract and the two recombinant proteins. Briefly, wells of 96 microtiter plates (Nunc) were coated overnight at 4°C with 200 μL of antigen solution (1 μg/mL of rEiSWP1 or 0.5 μg/mL of rEiPTP1 or 1 μg/mL of total protein extract) diluted in PBS buffer. Between each of the following steps, wells were washed three times in PBS– 0.05% Tween 20 (PBS-T). Wells were blocked with 300 μL of 5% (m/v) powdered milk in PBS-T at 37°C for 2 h. Then 300 μL/well of 1% of trehalose were added and plates were incubated at 37°C for 30 min. Plates were then stored at 4°C until use. For ELISA assay, 150 μL of plasmas diluted at 1/500 in 1% powdered milk—1% Tween 20 –PBS were added in duplicate, and plates were incubated at 37°C for 1 h. Then 100 μL/well of horseradish peroxydase conjugated goat anti-human IgG diluted at 1/20000 or anti-human IgA diluted at 1/8000 (Bio-Rad) were added and incubated at 37°C for 30 min. The TMB chromogenic substrate (100 μL/well, Kem-En-Tec Diagnostics) was added and incubated for 10 min. The reaction was stopped with 0.2 M H_2_SO_4_. Optical density was measured at 450 and 620 nm. Plasma from a patient who suffered from an intestinal infection with *E*. *intestinalis* was used as a positive control. The result for each patient was the average of duplicates of the indexes between the patient’s value and that of the positive control.

### Statistical analysis

Sample size was estimated according to Cohen’s recommendations, which define effect size thresholds as: small (ES: 0.2), medium (ES: 0.5) and large (ES: 0.8, "grossly perceptible and therefore large"). More specifically, for comparisons between CRC patients and healthy controls, a minimum of 130 subjects per group was required to highlight differences greater than 0.4 effect size for a two-sided type I error at 5% and 90% power. For comparisons between CRC patients and healthy controls by sex (subgroup analysis), medium to large effect sizes can be reported with a two-sided type I error of 5% and satisfactory 80% statistical power. Continuous data are expressed according to their statistical distribution as median and interquartile range. The assumption of normality was analyzed using the Shapiro-Wilk test. First, age was treated as continuous variable. Then, age was categorized according to statistical distribution and clinical/biological relevance. Student t-test or Mann-Whitney test were applied for comparisons between groups (such as CRC vs. controls). The homoscedasticity was studied using equality of variance Fisher-Snedecor test. The relationships between different ELISA tests were analyzed with Spearman correlation coefficient (ρ). Significance of the difference between two correlation coefficients was determined using the Fisher r-to-z transformation. A multivariate analysis was conducted with multiple logistic regression to take into account possible confounders (i.e. sex, age) in order to determine test or the combination of ELISA tests most associated with disease (CRC vs. controls). A subgroup analysis by sex was also performed to examine the relationships between ELISA tests and tumor characteristics, separately for women and men. A particular attention was also paid on the analysis of *sex x age* interaction. Lifestyle was appreciated considering that the inhabitants of towns with less than 2000 inhabitants had a rural lifestyle, while the inhabitants of towns with more than 2000 inhabitants had an urban life. Statistical analyses were performed using the Stata software package (version 15, StataCorp, College Station, US). All statistical tests were carried out for a two-sided type I error at 5%. No correction for multiple testing was applied except for the study of relationships between ELISA tests (Sidak’s type I error). However, results are expressed using effect-sizes (ES) and 95% confidence intervals and interpreted according to aforementioned Cohen’s recommendations.

## Results

### Preliminary evaluations

**Fig 1:**
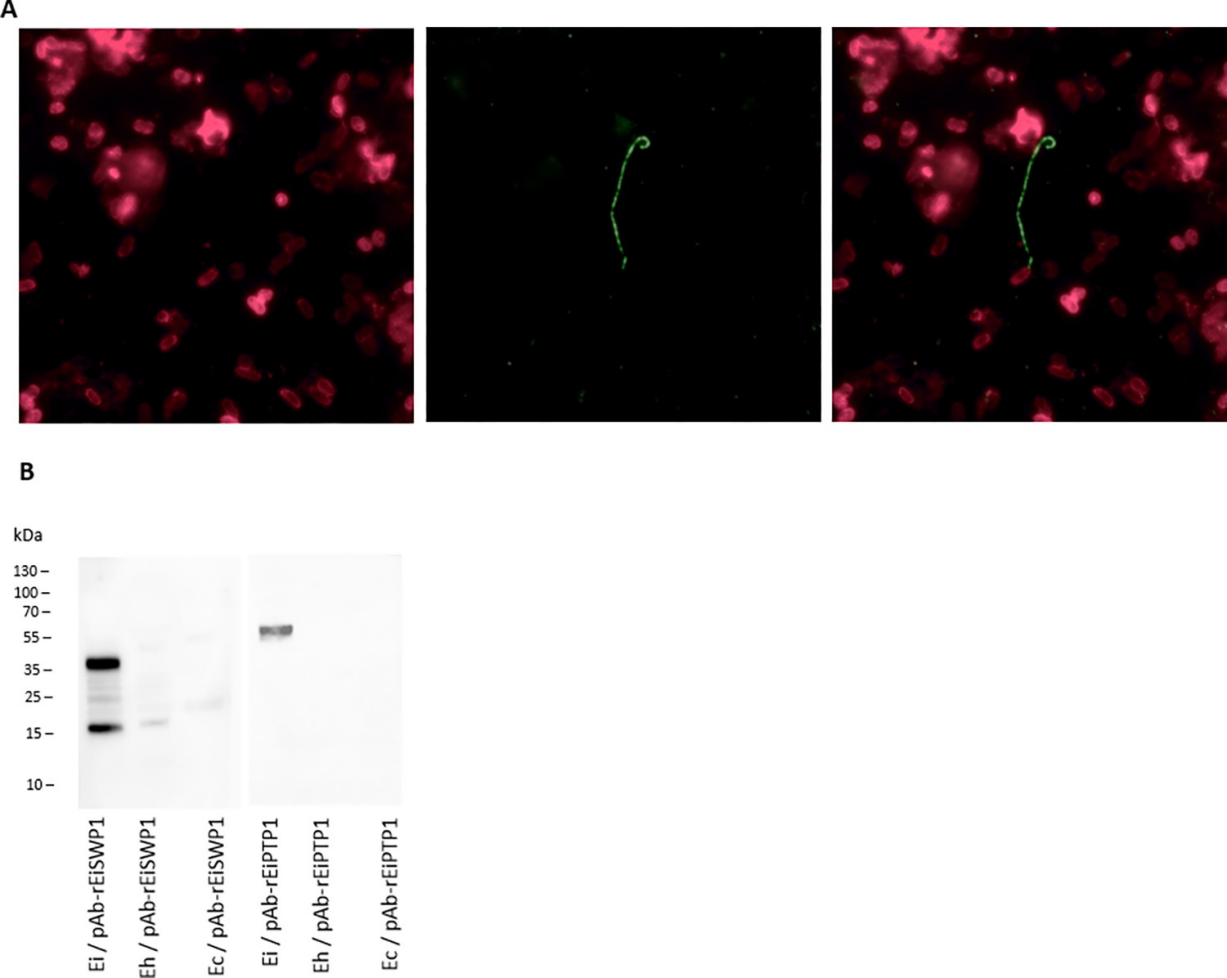
Evaluation of specificity using polyclonal antibodies pAb-rEiSWP1 and pAb-rEiPTP1 targeting rEiSWP1 and rEiPTP1, respectively. (A) IFAT labeling of PTP1 (green) and SWP1 (red) using pAb-rEiSWP1 and pAb-rEiPTP1 on *E*. *intestinalis* spores. (B) Western blot assay using pAb-rEiSWP1 and pAb-rEiPTP1 on *E*. *intestinalis* (Ei), *E*. *hellem* (Eh) and *E*. *cuniculi* (Ec) total protein extracts (expected size of EiSWP1 and EiPTP1 are 41.5 kDa and 69 kDa, respectively).

In order to assess the specificity of our rEiSWP1- and rEiPTP1-based ELISA assays, we immunized mice with both proteins to produce polyclonal antibodies (pAb-rEiSWP1 and pAb-rEiSWP1). As expected, IFAT assays performed on *E*. *intestinalis* spores showed a specific labeling of spore wall with pAb-rEiSWP1 and of the polar tube with pAb-rEiPTP1 (Fig 1A). Cross-reactivity with *E*. *hellem* and *E*. *cuniculi* was tested by Western blot. On *E*. *intestinalis* total protein extract, pAb-rEiSWP1 and pAb-rEiPTP1 labeled proteins corresponding to the expected sizes of EiSWP1 (41.5 kDa) and EiPTP1 (69 kDa). No cross-reactivity was observed with *E*. *hellem* or *E*. *cuniculi* (Fig 1B).

In order to verify the ELISA results with another method, IFAT were performed with the positive and negative plasma controls used to perform ELISA assays. The positive controls were responsible for labelling of *E*. *intestinalis* spores, whereas negative controls were not (S1 Fig).

### Description of human populations

The study population included 141 colorectal cancer (CRC) patients and 135 control subjects (*i*.*e*. patients free of cancer), with a sex ratio of 1.27 and 1.25, respectively (Table 1). The median age was 72 years for both groups. The proportion of individuals living in rural or urban areas was not significantly different between the two groups (p = 0.98).

The main data associated with cancer are reported in Table 1.

**Table 1:** Characteristics of patients included in the study.

	CRC group (n = 141)	Control group (n = 135)
Age (years)	72.1 ± 11.7	71.5 ± 14.5
Male	79/141 (56.0)	75/135 (55.6)
Tumor location		
Right colon	69/141 (48.9)	/
Left colon	63/141 (44.7)	/
Ileo-caecal	6/141 (4.3)	/
Small intestine	3/141 (2.1)	/
Chemotherapy before surgery	35/140 (25.0)	/
TNM classification (13)		
T0	7/125 (5.6)	/
T1	10/125 (8.0)	/
T2	17/125 (13.6)	/
T3	76/125 (60.8)	/
T4	15/125 (12.0)	/
N0	0/125 (0.0)	/
N1	1/125 (0.8)	/
N2	2/125 (1.6)	/
Nx	3/125 (2.4)	/
M0	31/124 (25.0)	/
M1	16/124 (12.9)	/
Mx	77/124 (62.1)	/
Metastases		
No	99/140 (70.7)	/
Yes	41/140 (29.3)	/
Relapse	19/141 (13.5)	/
Death	16/139 (11.5)	/

Data are presented as mean ± standard deviation or as number of cases / number of available data (percentage).

### *E*. *intestinalis* seroprevalence among CRC and control subjects

**Fig 2:**
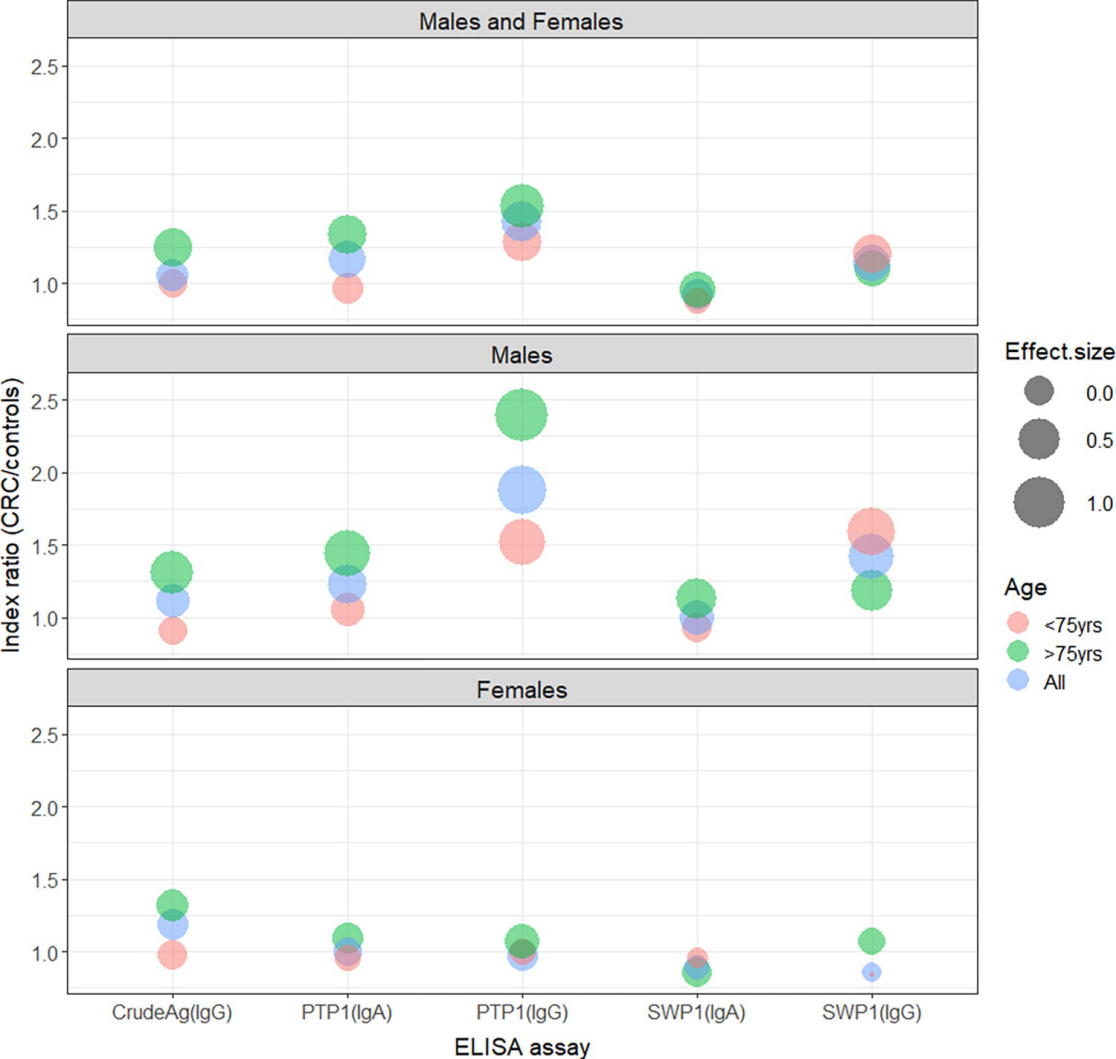
Effect-sizes according to the different antigens (abscissa) and ratio of indexes (DO patient/DO positive control) between CRC and controls (ordinates).

Overall, five different ELISA assays were performed: IgG/ crude antigens of *E*. *intestinalis*, IgG/rEiSWP1, IgG/rEiPTP1, IgA/rEiSWP1 and IgA/rEiPTP1. Concerning the entire population studied, patients with CRC had significantly higher anti-rEiPTP1 IgG levels than subjects in the control group (Tables 2 and 3). Anti-rEiSWP1 IgG levels tended to be higher, and to a lesser extent, anti-rEiPTP1 IgA levels also tended to be higher in the CRC group. Interestingly, when focusing only in men, these three markers (anti-rEiPTP1 IgG, anti-rEiSWP1 IgG, anti-rEiPTP1 IgA) were significantly increased in CRC compared to controls, but were not different between the two female groups (Table 3). Concerning age, the differences appeared more marked among the whole CRC population over 75 years old, and again, even more in men over 75 (Table 3). The largest effect-sizes were observed for anti-rEiPTP1 IgG in men (ES = 0.821 [0.492; 1.147]) and in men older than 75 years (ES = 1.01 [0.479; 1.535]) (Fig 2 and S1 Table).

**Table 2:** Odds-ratios of comparisons between CRC and control groups for each ELISA assay.

ELISA assay	OR [95% CI]
IgG total protein extract	1.16 [0.39; 3.44]
IgA anti-rEiSWP1	4.07 [1.79; 9.24]
IgG anti-rEiSWP1	1.72 [1.02; 2.89]
IgA anti-rEiPTP1	2.62 [0.86; 8.00]
IgG anti-rEiPTP1	1.06 [0.95; 1.17]

CI: confidence interval

**Table 3:** Median values of indexes for the different ELISA assays according to patient groups.

		Number of subjects	ELISA				
			IgG total protein extract	IgA anti-rEiSWP1	IgG anti-rEiSWP1	IgA anti-rEiPTP1	IgG anti-rEiPTP1
**All population study**							
	CRC	141	0.20 [0.15; 0.29]	1.70 [1.04; 2.76]	0.73 [0.49; 1.10]	0.28 [0.20; 0.40]	0.37 [0.22; 0.67]
	Controls	135	0.19 [0.13; 0.26]	1.84 [1.25; 2.58]	0.64 [0.42; 0.91]	0.25 [0.18; 0.38]	0.26 [0.16; 0.41]
	*p-value*	*/*	*0*.*121*	*0*.*590*	*0*.*067*	*0*.*088*	***<0*.*001***
**Males**							
	CRC	79	0.22 [0.15; 0.30]	1.93 [1.26; 3.25]	0.82 [0.53; 1.23]	0.32 [0.22; 0.48]	0.45 [0.22; 0.79]
	Controls	75	0.19 [0.15; 0.28]	1.94 [1.31; 2.95]	0.58 [0.41; 0.82]	0.26 [0.18; 0.41]	0.24 [0.15; 0.37]
	*p-value*	*/*	*0*.*455*	*0*.*760*	***<0*.*001***	***0*.*020***	***<0*.*001***
**Females**							
	CRC	62	0.19 [0.15; 0.28]	1.51 [0.91; 2.15]	0.64 [0.41; 0.91]	0.25 [0.19; 0.34]	0.33 [0.19; 0.49]
	Controls	60	0.16 [0.12; 0.25]	1.70 [1.22; 2.43]	0.75 [0.44; 1.21]	0.25 [0.18; 0.35]	0.34 [0.18; 0.56]
	*p-value*	*/*	*0*.*135*	*0*.*235*	*0*.*264*	*0*.*988*	*0*.*894*
**Age < 75 years**							
	CRC	82	0.21 [0.14; 0.29]	1.68 [1.04; 2.64]	0.75 [0.45; 1.12]	0.26 [0.20; 0.39]	0.32 [0.19; 0.60]
	Controls	77	0.21 [0.15; 0.28]	1.91 [1.28; 2.98]	0.62 [0.41; 0.86]	0.27 [0.18; 0.42]	0.25 [0.16; 0.39]
	*p-value*	*/*	*0*.*847*	*0*.*303*	*0*.*106*	*0*.*907*	***0*.*045***
**Age > 75 years**							
	CRC	59	0.20 [0.15; 0.33]	1.75 [1.03; 2.99]	0.73 [0.51; 1.07]	0.31 [0.19; 0.46]	0.46 [0.27; 0.77]
	Controls	58	0.16 [0.13; 0.23]	1.82 [1.25; 2.43]	0.66 [0.46; 0.96]	0.23 [0.15; 0.33]	0.30 [0.16; 0.46]
	*p-value*	*/*	***0*.*006***	*0*.*717*	*0*.*340*	***0*.*021***	***0*.*003***
**Males < 75 years**							
	CRC	48	0.21 [0.14; 0.30]	1.88 [1.11; 2.97]	0.92 [0.48; 1.35]	0.29 [0.21; 0.44]	0.38 [0.22; 0.77]
	Controls	45	0.23 [0.16; 0.29]	2.03 [1.51; 3.24]	0.58 [0.41; 0.81]	0.28 [0.18; 0.46]	0.25 [0.17; 0.39]
	*p-value*	*/*	*0*.*661*	*0*.*544*	***0*.*001***	*0*.*661*	***0*.*004***
**Males > 75 years**							
	CRC	31	0.22 [0.15; 0.33]	2.06 [1.41; 3.77]	0.70 [0.56; 1.12]	0.35 [0.24; 0.50]	0.55 [0.22; 0.93]
	Controls	30	0.17 [0.14; 0.23]	1.83 [1.31; 2.58]	0.59 [0.42; 0.84]	0.24 [0.15; 0.32]	0.23 [0.15; 0.31]
	*p-value*	*/*	***0*.*047***	*0*.*254*	*0*.*081*	***0*.*002***	***<0*.*001***
**Females < 75 years**							
	CRC	34	0.20 [0.14; 0.28]	1.63 [0.88; 2.20]	0.58 [0.43; 0.81]	0.26 [0.20; 0.30]	0.26 [0.16; 0.46]
	Controls	32	0.20 [0.13; 0.25]	1.70 [1.18; 2.71]	0.69 [0.41; 1.04]	0.27 [0.19; 0.36]	0.26 [0.16; 0.62]
	*p-value*	/	*0*.*778*	*0*.*376*	*0*.*254*	*0*.*729*	*0*.*748*
**Females > 75 years**							
	CRC	28	0.19 [0.15; 0.33]	1.47 [0.96; 2.12]	0.83 [0.36; 1.05]	0.24 [0.18; 0.35]	0.39 [0.28; 0.52]
	Controls	28	0.14 [0.11; 0.24]	1.72 [1.24; 2.28]	0.77 [0.48; 1.28]	0.22 [0.18; 0.34]	0.37 [0.25; 0.51]
	*p-value*	/	*0*.*053*	*0*.*427*	*0*.*831*	*0*.*909*	*0*.*854*

Data are presented as median [25^th^; 75^th^ percentiles] of the indexes between the patient’s DO value and that of the positive control. *p-values* in bold are significant (*p* < 0.05).

Regarding the other information available for CRC patients, antibody levels were not significantly different (S2 Table), except for anti-rEiSWP1 and deaths, particularly among women (index values of 0.61 and 1.11, p = 0.008, for alive and dead women, respectively).

### Correlation between ELISA assays

**Fig 3:**
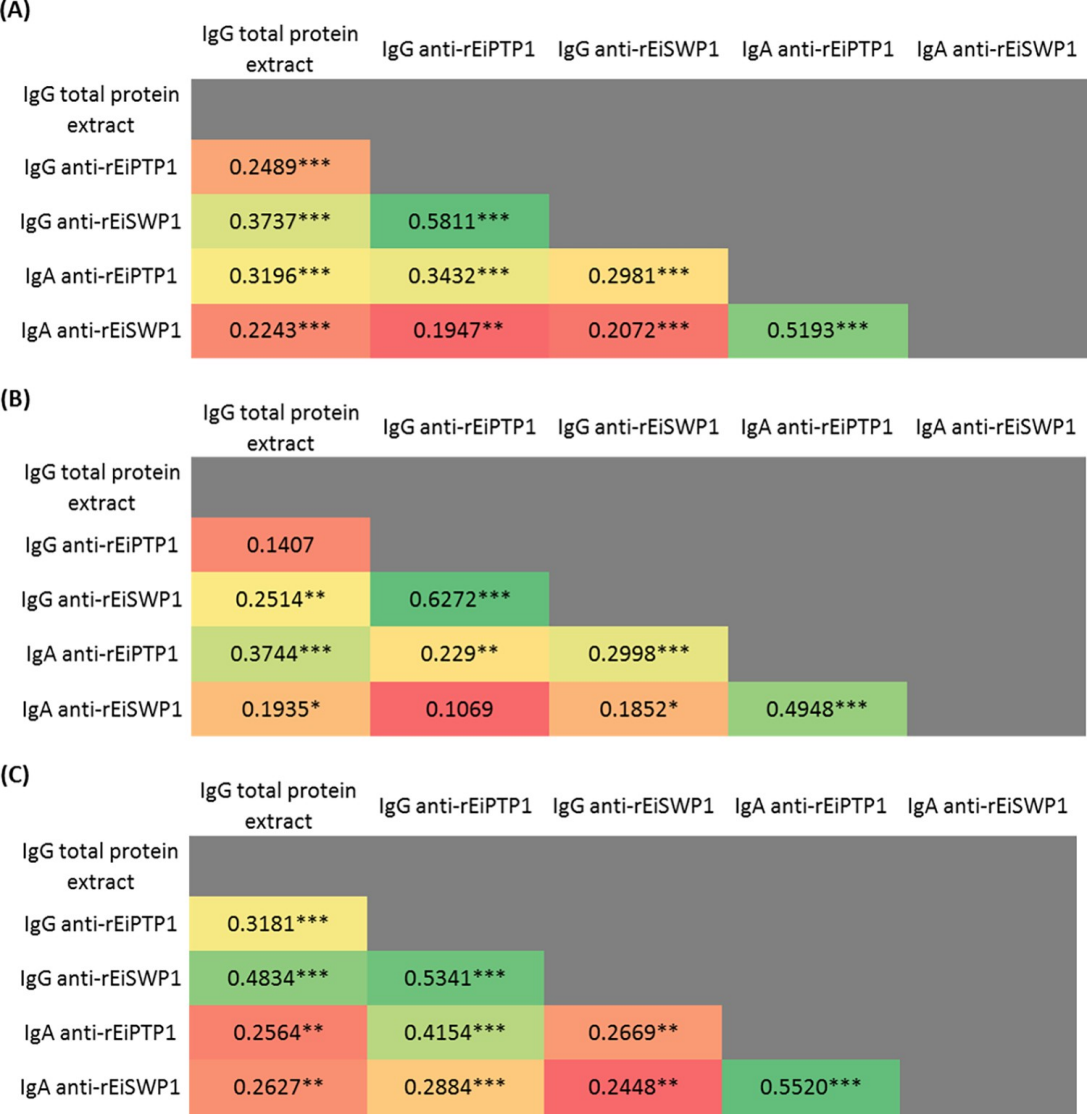
**Heat maps representing correlation coefficients (ρ) from results obtained with the different antigens within (A) global population, (B) controls and (C) CRC patients.** Within each table, a gradation from red to green represents an increasingly strong correlation. *p-values*: * < 0.05; ** < 0.01; *** < 0.001.

The correlation coefficient ρ was calculated for each antigens pair index of ELISA assays (Fig 3). ELISA assays based on rEiSWP1 and rEiPTP1 were the most correlated, whether for the detection of IgG on the one hand (ρ = 0.5811, 0.6272 and 0.5341, for the global population, for controls and for CRC patients, respectively) or IgA on the other hand (ρ = 0.5193, 0.4948 and 0.5520). The IgA results were poorly correlated with those of IgG, and vice versa. The differences between correlation coefficients found in patients and controls were not significant (z = 1.16 for IgG and 0.65 for IgA). Thus, correlation between the different assays was independent of the clinical status of the patients (CRC or controls).

### Multivariate analysis

No significant association was identified between antibodies levels detected by our ELISA assays, gender and age, and disease, except for IgA anti-rEiPTP1 among males over 75 years old (S3 Table).

## Discussion

Measuring exposure to infectious agents is most often done by detecting antibodies which are generally stable markers of exposure. In this way, serology is a simple macroscopic approach to describe an epidemiological link between infectious agents and chronic diseases, such as cancer [16,17]. However, this approach is of course not sufficient to rule on a causal link. Antibody detection is now frequently performed by ELISA, which can be designed from a crude lysate of the pathogen and/or recombinant proteins. The choice of antigens is a key step [18]. Working on crude lysates may lack sensitivity because the immunogenic antigens may be in low quantity within the total extract, whereas if the target protein is well chosen, an ELISA based on recombinant proteins will be more sensitive. For the development of an ELISA assay targeting *E*. *intestinalis*, we chose to particularly target PTP1 and SWP1. Indeed, SWPs are the first proteins encountered by an infected subject, and therefore are an interesting target for inducing an immune response following an infection, and strong antigenicity of PTP, and particularly PTP1, has already been reported several times [8,9,19–21]. Furthermore, the production of antibodies against SWP1 and PTP1 would not follow the same kinetics. As was demonstrated in a man infected with *E*. *cuniculi*, the first antibodies that appear would be directed against the spore wall [8]. The later appearance of anti-PTP antibodies has also been described in experimentally infected mice [22]. Thus our ELISAs make it possible to identify individuals who have been immunized for a more or less long time. Then, it has been shown that specific antibodies persist for at least 6 years after infection with *E*. *cuniculi* [8]. Finally, the combination of the results of these 2 antigens allows to strengthen our results, as demonstrated by the significant correlation factors we obtained between rEiSWP1 and rEiPTP1 indexes obtained by ELISA. This correlation was independent of the clinical status (CRC or controls) of patients. IFAT observations confirmed that both recombinant proteins correspond to proteins expressed by *E*. *intestinalis* and are localized as expected. Moreover, the specificity of the labeling using polyclonal antibodies on *E*. *intestinalis* spores confirmed that the strong correlation between rEiSWP1 and rEiPTP1 ELISA assays was not due to cross-reactivity between targets and should therefore be considered confirmation of true prior contact with microsporidia. It was previously shown that wall protein extracts for serological assays lack specificity in distinguishing between *Encephalitozoon* species. On the other hand, no or only little cross-reactivity between *Encephalitozoon* species was observed with polar tube proteins [8,23,24]. Variability in the amino acid sequences of the internal region of PTP1 could explain these differences in cross-reactivity [25]. In our study, we did not observe positive reaction (i) by Western blot with pAb-rEiSWP1 or pAb-anti-rEiPTP1 on protein extract from *E*. *hellem* or *E*. *cuniculi*; (ii) from the ELISA targeting EiSWP1 using sera from patients infected with *E*. *hellem*. Of note, we previously reported that *E*. *hellem* infections were as common as *E*. *intestinalis* infections, so it would be interesting to develop an ELISA targeting specifically this species [2].

Due to the disturbances induced in the intestinal cells they infect (reviewed in [12]), it can be speculated that microsporidia could be involved in the occurrence of CRC. Colorectal cancer tumor development occurs following the accumulation of genetic mutations in colonic or rectal cells over the course of the individual’s life. But it has also been shown that the interactions of cells with their microenvironment, including the intestinal microbiota, could modulate the development capacities of the tumor [26,27]. Furthermore, some eukaryotic pathogens, such as *Cryptosporidium parvum*, a cosmopolitan intracellular parasite responsible for diarrhea, could also participate in the appearance of CRC [28]. In the present study, we observed a significantly higher level of anti-*E*. *intestinalis* antibodies in CRC patients compared to subjects without cancer, with some differences depending on the targeted antigens and immunoglobulin isotypes. As explained above, the particular immunogenicity of PTP1 may explain why our observations were more significant with antibodies targeting this protein, compared to crude lysate or even SWP1. The differences we observed were particularly marked in the male population, which could suggest a different pathophysiology of CRC and/or a different susceptibility to microsporidia infection between men and women. We observed also higher antibody levels in older individuals (> 75 years old) and, again, particularly among men suffering from CRC. The reasons for these higher levels of anti-*E*. *intestinalis* antibodies in men and elderly people with CRC are obscure, but the impact of these parameters on the appearance of a disease following contamination by an infectious agent, but also on the process of oncogenesis following an infection, has already been described. For example, intestinal infection with *Entamoeba histolytica* leads to hepatic amebiasis, which is more common in males. It was thus shown in mice that androgens led to reduced activation and maturation of neutrophils, and therefore to prolonged survival of amoebic trophozoites, leading to a deleterious cycle contributing to the destruction of the liver [29]. Moreover, during hepatitis B virus (HBV) infection, the host’s inflammatory immune response to the viral antigens induces hepatocyte damage and is followed by the pathogenesis of liver cancer, but sex hormones may interact with HBV infection in this process and lead to a dominant sex disparity in liver cancer risk [30]. Finally, factors intrinsic to the host (genetics, age, sex, etc.) associated or not with infection due to *Helicobacter pylori* carrying some virulence factors, and as well as environmental factors, influence the risk of gastric cancer [31]. These mechanisms deciphered for other pathogens provide avenues for research to explore a potential link between microsporidia and cancer.

We chose to search for IgG, reflecting previous contact and therefore potentially prior to the initiation of the cancerous phenomenon, but also IgA to demonstrate an immune response against an enteric pathogen. Higher anti-rEiPTP1 IgA levels in CRC patients support a continued exposure to these antigens which may suggest a failure of the patient to eliminate the pathogen. Redondo et *al*. detected *Encephalitozoon* DNA in intestinal tissues (tumoral and healthy peritumoral tissues) of 41.3% patients with CRC, but no cases among control subjects [12]. This raises questions because it suggests that these patients would present with diarrhea and microsporidia should be detected in their stools. We acknowledge that this is a limitation of our study, as we did not have access to stool or intestinal biopsies from our CRC patients.

Regarding severity factors (TNM classification, relapses,…), there is overall no characteristic element that correlates with the antibody levels that we detected. Interestingly, higher levels of anti-rEiSWP1 IgG were detected in deceased women. Even though this is an interesting result, especially since we did not observe any other change in antibody levels in women, further studies need to be conducted to confirm this result before it can be considered as a prognostic marker. Indeed, a gender effect on survival prognostic has already been reported in cancers associated with infectious agents. It has been described a synergistic effect of male sex and HBV infection (HBsAg positivity) on liver cancer mortality, particularly in younger people [32].

Among the limitations of our study, we cannot exclude that for certain CRC patients, due to their immunosuppression, serology may not be fully reliable. However, this impact would have reduced significance of our results and has then no impact on our conclusions. Moreover, in our cohort some patients underwent chemotherapy and we did not observe significant differences in antibody levels between these patients compared to CRC patients without chemotherapy. Then, it is also possible that CRC patients are at greater risk of microsporidiosis given the opportunistic potential of this pathogen, but we did not find such a diagnosis in their history. The retrospective design of the study is also a limitation since we do not have precise data on the risk of exposure of our patients to microsporidia. Concerning the lifestyle (rural or urban), by extrapolating on the number of inhabitants per municipality of residence, we observe no difference between the control group and the CRC group but it would be necessary to have information on travel, leisure, etc. Finally, we acknowledge that one limitation of our study was the use of controls patients and not healthy people without any history of hospitalization.

To conclude, our results highlight higher anti-*E*. *intestinalis* specific IgG and IgA levels in CRC patients. This could suggest a role for *E*. *intestinalis* in carcinogenesis, while some *in vitro* experimental data make this hypothesis credible. *In vivo* results using animal models are now necessary to go further in exploring the mechanisms involved. Moreover, data on the most common species in humans, *E*. *bieneusi*, are now needed.

## Supporting information

S1 Fig Immunofluorescence labelling of spores smears of *Encephalitozoon intestinalis*. Two plasma of patients (A and B) previously diagnosed with *E*. *intestinalis* microsporidiosis (*i*.*e*. positive controls) and two plasma of patients (C and D) without any history of this infection were tested with IFAT. With negative controls, only spore nuclei (arrows) are visible. Human immunoglobulins labellings appeared in green and DAPI labelling in blue.
(TIF)

S1 Table Effect-sizes according to ELISA assays and groups of patients. (DOCX)

S2 Table Median values of indexes for the different ELISA tests according to characteristics of tumors. Data are presented as median [25^th^; 75^th^ percentiles] of the indexes between the patient’s DO value and that of the positive control. *p-values* in red are significant (p < 0.05).
(XLSX)

S3 Table Multivariate analysis for the different ELISA assays according to gender and age group. Data presented are P>|t| [95% CI] and regression coefficient in italics. Data P>|t| in bold are significant (< 0.05).
(DOCX)
