# Supplementary figures and images for: Increased levels of anti-Encephalitozoon intestinalis antibodies in patients with colorectal cancer
Source: PLoS Negl Trop Dis. 2024 Sep 9;18(9):e0012459. doi: 10.1371/journal.pntd.0012459 (PMC11412658; doi:10.1371/journal.pntd.0012459)

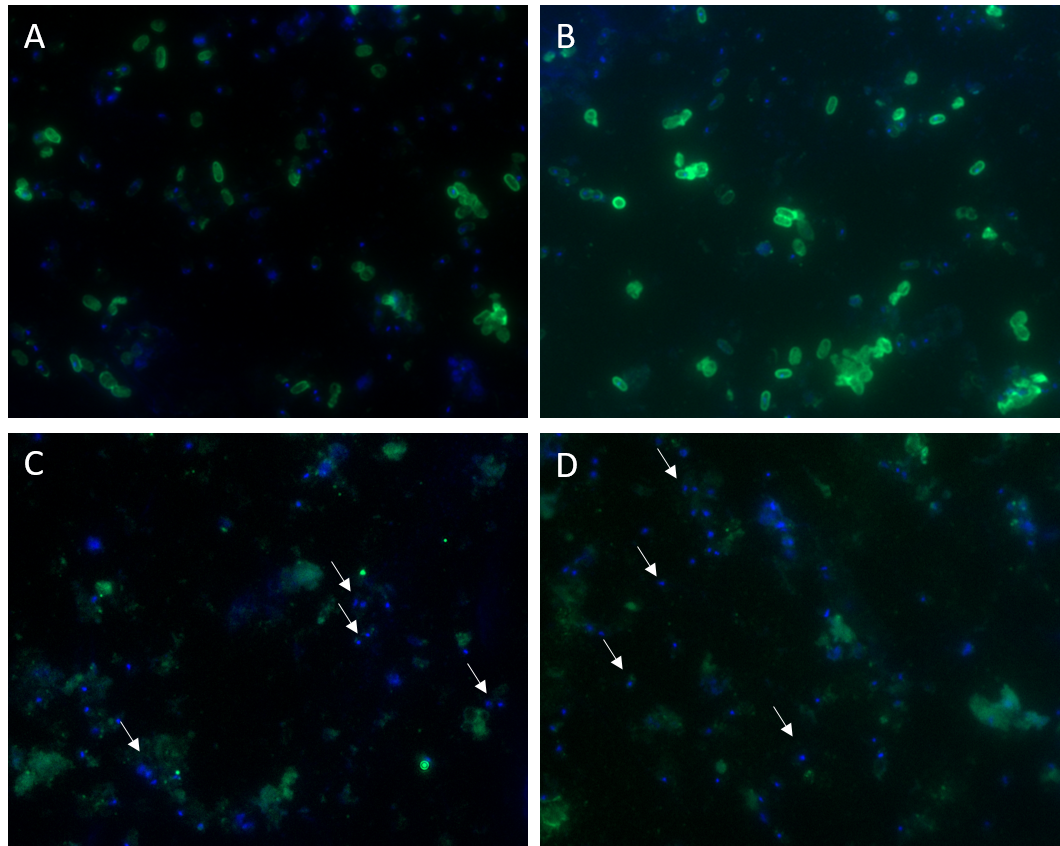

Supplement: S1 Fig — Two plasma of patients (A and B) previously diagnosed with E. intestinalis microsporidiosis (i.e. positive controls) and two plasma of patients (C and D) without any history of this infection were tested with IFAT. With negative controls, only spore nuclei (arrows) are visible. Human immunoglobulins labellings appeared in green and DAPI labelling in blue. (TIF) [file pntd.0012459.s001.tif]
