# Supplementary material for: Increased levels of anti-Encephalitozoon intestinalis antibodies in patients with colorectal cancer
Source: PLoS Negl Trop Dis. 2024 Sep 9;18(9):e0012459. doi: 10.1371/journal.pntd.0012459 (PMC11412658; doi:10.1371/journal.pntd.0012459)
Supplement: S1 Table — (DOCX) [file pntd.0012459.s002.docx]

**Table S1. Effect-sizes according to ELISA assays and groups of patients.**

| Group | ELISA | Effect-size | 95%CI inf | 95%CI sup |
| --- | --- | --- | --- | --- |
| All | total crude antigens IgG | -0.077 | -0.313 | 0.158 |
|  | anti-rEiSWP1 IgA | -0.035 | -0.271 | 0.200 |
|  | anti-rEiSWP1 IgG | -0.246 | -0.482 | -0.009 |
|  | anti-rEiPTP1 IgA | -0.215 | -0.451 | 0.021 |
|  | anti-rEiPTP1 IgG | -0.453 | -0.691 | -0.214 |
| Males | total crude antigens IgG | -0.103 | -0.418 | 0.211 |
|  | anti-rEiSWP1 IgA | -0.156 | -0.471 | 0.159 |
|  | anti-rEiSWP1 IgG | -0.652 | -0.973 | -0.328 |
|  | anti-rEiPTP1 IgA | -0.387 | -0.704 | -0.069 |
|  | anti-rEiPTP1 IgG | -0.821 | -1.147 | -0.492 |
| Females | total crude antigens IgG | -0.041 | -0.393 | 0.312 |
|  | anti-rEiSWP1 IgA | 0.123 | -0.231 | 0.475 |
|  | anti-rEiSWP1 IgG | 0.217 | -0.137 | 0.570 |
|  | anti-rEiPTP1 IgA | 0.03 | -0.323 | 0.382 |
|  | anti-rEiPTP1 IgG | -0.026 | -0.378 | 0.327 |
| All < 75years | total crude antigens IgG | 0.031 | -0.279 | 0.340 |
|  | anti-rEiSWP1 IgA | 0.100 | -0.210 | 0.410 |
|  | anti-rEiSWP1 IgG | -0.302 | -0.613 | 0.009 |
|  | anti-rEiPTP1 IgA | -0.043 | -0.352 | 0.267 |
|  | anti-rEiPTP1 IgG | -0.364 | -0.675 | -0.051 |
| All > 75 years | total crude antigens IgG | -0.26 | -0.621 | 0.103 |
|  | anti-rEiSWP1 IgA | -0.209 | -0.570 | 0.152 |
|  | anti-rEiSWP1 IgG | -0.173 | -0.534 | 0.188 |
|  | anti-rEiPTP1 IgA | -0.386 | -0.748 | -0.021 |
|  | anti-rEiPTP1 IgG | -0.567 | -0.933 | -0.199 |
| Males < 75 years | total crude antigens IgG | 0.050 | -0.354 | 0.453 |
|  | anti-rEiSWP1 IgA | 0.018 | -0.385 | 0.421 |
|  | anti-rEiSWP1 IgG | -0.759 | -1.175 | -0.339 |
|  | anti-rEiPTP1 IgA | -0.122 | -0.525 | 0.282 |
|  | anti-rEiPTP1 IgG | -0.683 | -1.096 | -0.266 |
| Males > 75 years | total crude antigens IgG | -0.535 | -1.038 | -0.028 |
|  | anti-rEiSWP1 IgA | -0.407 | -0.906 | 0.096 |
|  | anti-rEiSWP1 IgG | -0.469 | -0.970 | 0.035 |
|  | anti-rEiPTP1 IgA | -0.704 | -1.212 | -0.189 |
|  | anti-rEiPTP1 IgG | -1.010 | -1.535 | -0.479 |
| Females < 75 years | total crude antigens IgG | -0.003 | -0.48 | 0.474 |
|  | anti-rEiSWP1 IgA | 0.216 | -0.263 | 0.694 |
|  | anti-rEiSWP1 IgG | 0.356 | -0.126 | 0.836 |
|  | anti-rEiPTP1 IgA | 0.105 | -0.373 | 0.582 |
|  | anti-rEiPTP1 IgG | 0.113 | -0.365 | 0.590 |
| Females > 75 years | total crude antigens IgG | -0.075 | -0.592 | 0.442 |
|  | anti-rEiSWP1 IgA | 0.016 | -0.500 | 0.533 |
|  | anti-rEiSWP1 IgG | 0.083 | -0.434 | 0.599 |
|  | anti-rEiPTP1 IgA | -0.024 | -0.541 | 0.493 |
|  | anti-rEiPTP1 IgG | -0.151 | -0.668 | 0.367 |
