# Supplementary material for: Increased levels of anti-Encephalitozoon intestinalis antibodies in patients with colorectal cancer
Source: PLoS Negl Trop Dis. 2024 Sep 9;18(9):e0012459. doi: 10.1371/journal.pntd.0012459 (PMC11412658; doi:10.1371/journal.pntd.0012459)
Supplement: S3 Table — Data presented are P>|t| [95% CI] and regression coefficient in italics. Data P>|t| in bold are significant (< 0.05). (DOCX) [file pntd.0012459.s004.docx]

**Supplementary Table S3. Multivariate analysis for the different ELISA assays according to gender and age group.**

Data presented are P>|t| [95 % CI] and regression coefficient in italics. Data P>|t| in bold are significant (< 0.05).

|  | ELISA | | | | |
| --- | --- | --- | --- | --- | --- |
|  | IgG total protein extract | IgA anti-rEiSWP1 | IgG anti-rEiSWP1 | IgA anti-rEiPTP1 | IgG anti-rEiPTP1 |
| Males and age > 75 years | 0.217 [-0.034; 0.147]  *0.057* | 0.183 [-0.102; 0.529]  *0.214* | 0.352 [-0.220; 0.079]  *-0.070* | **0.031** [0.011; 0.212]  *0.111* | 0.165 [-0.040; 0.233]  *0.097* |
| Females and age > 75 years | 0.830 [-0.085 ; 0.106]  *0.010* | 0.586 [-0.247; 0.436]  *0.094* | 0.543 [-0.124; 0.234]  *0.055* | 0.765 [-0.085; 0.115]  *0.015* | 0.452 [-0.098; 0.219]  *0.060* |
